# British and Foreign Medico-Chirurgical Review

**Published:** 1850-10

**Authors:** 


*British and Foreign Medico-Chirurgical Review.* July, 1850. *American Edition.* New York: R. & G. S. Woods.

We have received the American reprint of this valuable periodical, which is offered to the profession in this country at the moderate rate of three dollars per annum.

